# Immunotherapy – 2073. Therapeutic effect of mite allergen vaccines by subcutaneous route in allergic Cuban asthmatic patients

**DOI:** 10.1186/1939-4551-6-S1-P155

**Published:** 2013-04-23

**Authors:** Raúl Lázaro Castro Almarales, Mercedes Ronquillo Díaz, Mirta Alvarez Castello, Mayda González León, Alexis Labrada Rosado, José Severino Rodríguez Canosa, Maytee Mateo Morejón, Yunia Oliva Diaz, Bárbara Ivonne Navarro Viltres

**Affiliations:** 1 Allergens Department, National Center of Bioproducts (BIOCEN), Allergology and General Integral Medicine, La Habana, Cuba; 2 Allergen Service, University Hospital "General Calixto García", Cuba; 3 Allergology Department, University Hospital General Calixto García, Havana, Cuba; WAO Member; Cuban Society of Allergy, Asthma and Clinical Immunology, Member; Cuban Society of Immunology Member; 4 Docent Polyclinic "Pedro Fonseca", Havana, Cuba; Cuban Society of Integral General Medicine, Member; Cuban Society of Allergy, Asthma and Clinical Immunology, Member; Cuban Society of Immunology Member; 5 Cuban Society of Allergy, Asthma and Clinical Immunology, Mayabeque, Cuba; 6 Allergen Service, University Hospital "General Calixto García", Havana, Cuba; WAO Member; Cuban Society of Allergy, Asthma and Clinical Immunology, Member; Cuban Society of Immunology Member; 7 Allergen, Biocen, Cuba; 8 Allergen Department, National Center of Bioproducts (BIOCEN), Mayabeque, Cuba; Cuban Society of Immunology, Member; Cuban Society of Allergy, Asthma and Clinical Immunology, Member; 9 Docent Polyclinic "Federico Capdevila", Havana, Cuba; WAO Member; Cuban Society of Allergy, Asthma and Clinical Immunology, Member

## Background

World Health Organization position paper on immunotherapy recommends using mite allergen vaccines for rhinitis and mild to moderate asthma. Allergic sensitization to domestic mites and particularly to *Dermatophagoides pteronyssinus*, *Dermatophagoides siboney* and *Blomia tropicalis* has been described before in Cuba, as strongly linked to respiratory allergy symptoms. The aim of this work was to study the therapeutic effect and safety of allergen vaccines of these 3 mite species (VALERGEN, BIOCEN, Cuba) by subcutaneous route, in asthmatic patients.

## Methods

Three Double-Blind Placebo-Controlled clinical trials were performed in 40 patients each, showing asthmatic symptoms and positive predominant Skin Prick Test (SPT) to each mite, respectively. Half of patients received the active treatment consisting of subcutaneous injections with increasing doses, up to 6000 BU. Therapeutic effect was assessed after 6 and 12 months using symptoms/medication diary cards, peak expiratory flow (PEF) measures and skin sensitivity to investigated mites. Adverse reactions were classified using the World Allergy Organization scale.

## Results

The total 1 year cumulative dose was 63035 BU, in an average of 20.5 injections. The treatment was effective in the reduction of clinical symptoms (up to 32%, 95%CI:28-36%; p=0.0006) and medication intake (23%, 95%CI:18-28%), as compared to control treatment. The skin sensitivity to the allergens decreased significantly (p=0.0001), with regard to the beginning of the treatment. The allergen amount needed to induce a positive SPT increased 297-fold. Improvement of the lung function was observed, expressed in a modest Peak-Expiratory-Flow increase (p<0.05) and reduction of PEF daily variability. SIT was considered effective in 71% of patients. The frequency of local adverse reactions was 2.4 % of injections.

## Conclusions

Summing up subcutaneous immunotherapy, using VALERGEN vaccines, is effective and safe for the control and amelioration of the asthma.

